# Cerebral air embolism after implantation of a leadless pacemaker via the right internal jugular vein: a case report

**DOI:** 10.1093/ehjcr/ytag446

**Published:** 2026-06-09

**Authors:** Pierre Ollitrault, Mayane Al Khoury, Laure Champ-Rigot, Jonaz Font, Arnaud Pellissier

**Affiliations:** Department of Cardiology; Caen University Hospital, Normandie Univ, UniCaen, Avenue de la Côte de Nacre, Caen F-14000, France; Normandy Heart Rhythm Association, Avenue de la Côte de Nacre, Caen F-14000, France; Department of Cardiology; Caen University Hospital, Normandie Univ, UniCaen, Avenue de la Côte de Nacre, Caen F-14000, France; Normandy Heart Rhythm Association, Avenue de la Côte de Nacre, Caen F-14000, France; Department of Cardiology; Caen University Hospital, Normandie Univ, UniCaen, Avenue de la Côte de Nacre, Caen F-14000, France; Normandy Heart Rhythm Association, Avenue de la Côte de Nacre, Caen F-14000, France; Department of Cardiology; Caen University Hospital, Normandie Univ, UniCaen, Avenue de la Côte de Nacre, Caen F-14000, France; Normandy Heart Rhythm Association, Avenue de la Côte de Nacre, Caen F-14000, France; Department of Cardiology; Caen University Hospital, Normandie Univ, UniCaen, Avenue de la Côte de Nacre, Caen F-14000, France; Normandy Heart Rhythm Association, Avenue de la Côte de Nacre, Caen F-14000, France

**Keywords:** Leadless pacemaker, Internal jugular vein, Safety, Cerebral air embolism, Case report

## Abstract

**Background:**

Leadless pacemaker (LP) implantation is associated with fewer device-related complications than conventional transvenous pacing systems. While traditionally performed via the femoral vein, the right internal jugular (RIJ) approach has recently emerged as an alternative strategy, potentially improving catheter control. However, safety data for this approach remain limited.

**Case summary:**

A 77-year-old man with precapillary pulmonary hypertension, diabetes mellitus, and recurrent traumatic syncope underwent LP implantation via the RIJ vein following documentation of infra-Hissian conduction delay (HV interval 80 ms). The procedure was uneventful, with satisfactory electrical parameters. Immediately after repositioning the patient in a 30° head-up position, he developed acute neurological deterioration (Glasgow Coma Scale 6/15). Transthoracic echocardiography (TTE) excluded pericardial effusion but revealed air bubbles in the right ventricle. Cerebral computed tomography demonstrated multiple air bubbles within the right intracerebral venous system, consistent with retrograde cerebral venous air embolism. Emergency management included Trendelenburg positioning, 100% oxygen administration, and hyperbaric oxygen therapy. Neurological status improved, although a persistent left-sided motor deficit remained. At 15-month follow-up, no further syncope occurred, and the LP functioned normally, but residual left limbs hypokinesia persisted.

**Discussion:**

This case illustrates a rare but severe complication of RIJ LP implantation. Large-bore sheath use, low venous pressure in the jugular system, relative dehydration, severe pulmonary vascular disease, and early head-up positioning likely contributed to retrograde cerebral venous air migration. Strict preventive measures—including meticulous system flushing, adequate hydration, maintenance of slight Trendelenburg positioning, and post-procedural echocardiographic screening—may help mitigate this risk.

Learning points

- The right internal jugular approach for leadless pacemaker implantation may rarely cause severe complications such as retrograde cerebral venous air embolism.
- Meticulous flushing, adequate hydration, slight Trendelenburg positioning, and careful post-procedural management are essential to prevent air embolism during jugular leadless pacemaker implantation.

## Introduction

In patients requiring permanent pacing, implantation of a leadless pacemaker (LP) has been associated with a lower risk of procedural and device-related complications compared with conventional transvenous systems.^1,2^ Historically implanted via the femoral venous approach, LPs can now also be delivered through the right internal jugular vein (RIJ), with the aim of further reducing procedural complications and potentially improving catheter control.^3–5^ However, data regarding the safety of this approach as a first-line strategy across all patient profiles remain limited.

We report a case of RIJ vein LP implantation complicated by a serious postoperative retrograde cerebral air embolism in a patient with multiple comorbidities.

## Summary figure

**Figure:**
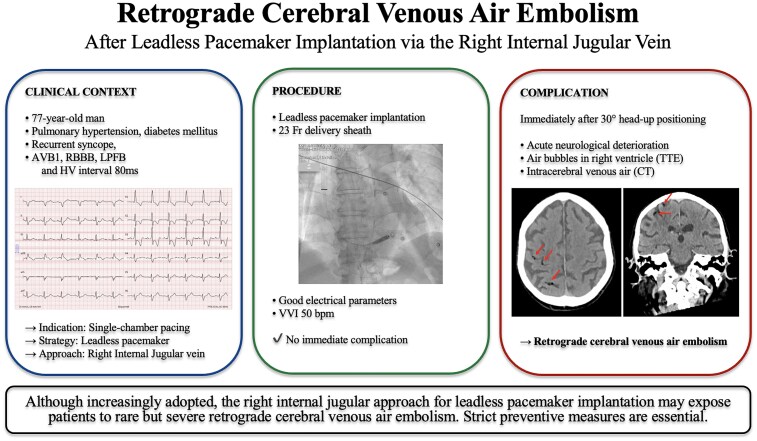
Abbreviations: AVB1, first degree atrioventricular block; CT, computed tomography; HV, His-to-ventricle; LPFB, left posterior fascicular block; RBBB, right bundle branche block; TTE, transthoracid echocardiography.

## Case presentation

A 77-year-old man was admitted to the cardiac intensive care unit for recurrent traumatic syncopal episodes. His medical history was notable for precapillary pulmonary hypertension related to veno-occlusive disease (treated with ambrisentan and tadalafil), chronic *cor pulmonale* without right ventricular systolic dysfunction, type 2 diabetes mellitus, and chronic alcohol abuse. He was receiving long-term oxygen therapy (3 L/min via nasal cannula). Syncope occurred at rest, except for one episode during ambulation that resulted in a scalp laceration requiring suturing. The admission 12-lead electrocardiogram showed sinus rhythm at 70 b.p.m., first-degree atrioventricular block (PR 200 ms), right bundle branch block (QRS 137 ms), and right axis deviation suggestive of associated left posterior fascicular block (*Figure 1*). TTE demonstrated stable findings compared with previous examinations: preserved left and right ventricular ejection fractions, cardiac index 2.5 L/min/m^2^, right ventricular dilatation with paradoxical septal motion, systolic pulmonary artery pressure estimated at 45 mmHg, and peak tricuspid regurgitation velocity of 3.1 m/s. Forty-eight hour telemetry monitoring revealed no high-grade conduction disturbances. An invasive electrophysiological study performed via the right femoral vein demonstrated a prolonged HV interval of 80 ms. According to current guidelines, single-chamber ventricular pacing was indicated,^6^ and given the elevated infectious risk (diabetes mellitus), a LP was preferred.^7^

**Figure 1:**
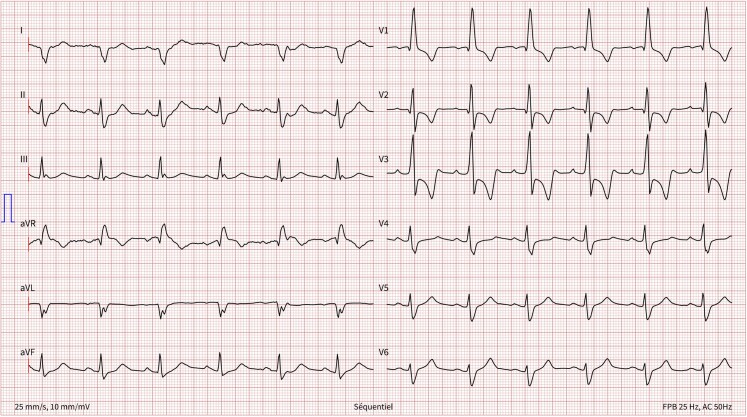
Twelve-lead electrocardiogram at admission.

LP implantation (Micra VR^TM^, Medtronic, USA) was performed via the RIJ vein under mild sedation using standard procedural techniques (*Figure 2*). The device was successfully positioned on the right ventricular septum without immediate complications. Electrical parameters were satisfactory (threshold 0.25 V at 0.24 ms, sensing >20 mV, impedance 1050 Ω) and the device was programmed in VVI mode at 50 bpm.

**Figure 2:**
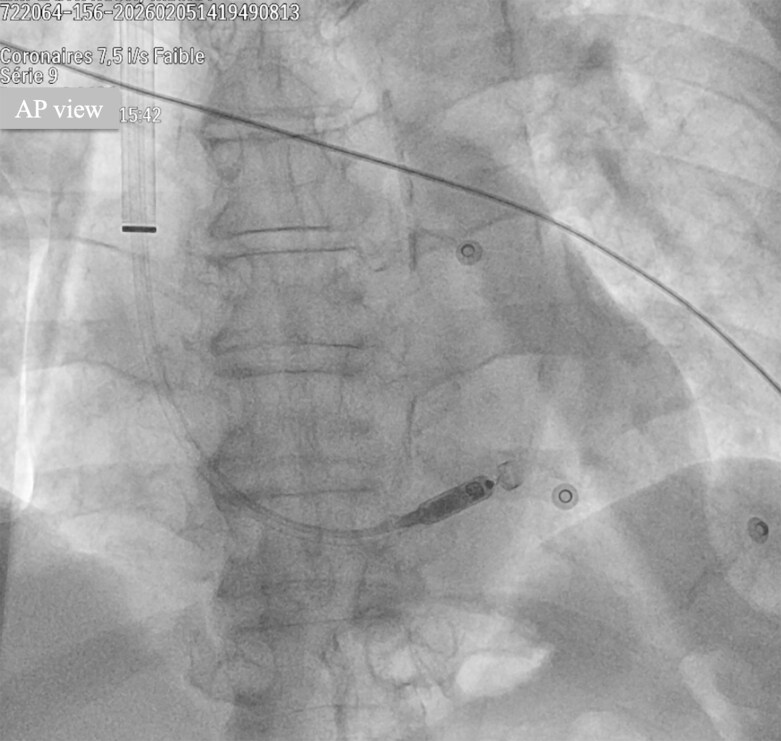
Implantation of a leadless pacemaker on the right ventricular septum, via the right internal jugular vein, in anterior-posterior fluoroscopy view.

Immediately post-procedure, after transferring the patient in bed with a proclive 30° position, he developed acute neurological deterioration with a Glasgow Coma Scale of 6/15 and leftward head deviation. Haemodynamic and respiratory parameters remained stable. TTE revealed no evidence of pericardial effusion but demonstrated the presence of air bubbles within the right ventricle. Emergency cerebral and supra-aortic CT angiography showed no arterial stenosis or occlusion of the supra-aortic trunks, circle of Willis, or intracranial arteries. Perfusion imaging was normal. However, multiple air bubbles were identified within the right intracerebral venous structures (*Figure 3*), consistent with retrograde cerebral venous air embolism.

**Figure 3:**
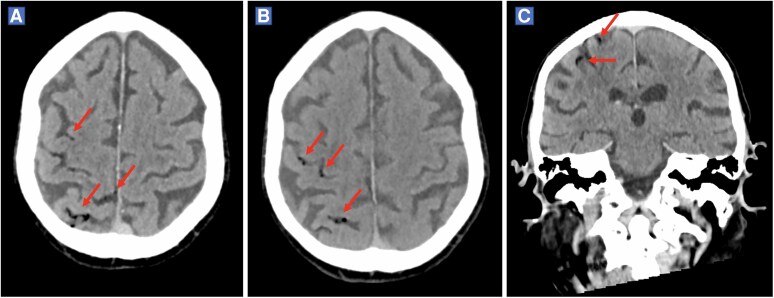
Cerebral and supra-aortic CT angiography. Multiple air bubbles (red arrows) are identified within the right intracerebral venous structures, in axial (*A*, *B*) and coronal (*C*) views.

Management was initiated immediately: the patient was placed in Trendelenburg position and received 100% oxygen therapy followed by hyperbaric oxygen treatment. Neurological status progressively improved to a Glasgow Coma Scale of 15, although a persistent left-sided motor deficit remained. After stabilization, he was transferred to a neurological rehabilitation centre. Bubble contrast TTE found no evidence of right-to-left shunt.

At 15-month follow-up, there had been no recurrence of syncope. The 12-lead ECG was unchanged. The LP functioned normally, with 19% right ventricular pacing in VVI 50 b.p.m. mode. Neurological rehabilitation resulted in partial motor recovery, but persistent hypokinesia of the left limbs remained, leading to significant functional impairment.

## Discussion

LP implantation is associated with lower rates of procedural and long-term complications compared with conventional transvenous pacing systems.^1,2^ While traditionally performed via the femoral vein, recent reports suggest that the RIJ approach may further improve procedural workflow and safety.^3–5^ To our knowledge, this is the first reported case of severe retrograde cerebral venous air embolism following LP implantation via the RIJ vein.

Air embolism is a rare but recognized complication of procedures involving central venous access (including conventional pacemaker/defibrillator implantation, catheter ablation, central venous catheterization, haemodialysis catheter placement, interventional radiology procedures, contrast injection, etc.) as well as surgery performed in semi-sitting positions.^8,9^ It’s true incidence during pacemaker implantation is difficult to estimate, as a proportion of cases may remain clinically silent.^10^

In this patient, several factors likely contributed to the occurrence of this complication. LP implantation requires the use of a large-bore delivery sheath (23 Fr internal diameter), which may facilitate venous air entry. Incomplete flushing of irrigation lines could also have contributed. Additionally, the internal jugular venous system is a low-pressure circuit, potentially favouring air aspiration. Pre-procedural fasting and relative dehydration may have reduced central venous pressure, increasing susceptibility to air aspiration. The severity of the clinical presentation was also multifactorial. Severe pulmonary vascular disease likely reduced pulmonary clearance of air bubbles within the right-sided circulation. The retrograde cerebral venous migration of air was probably facilitated by the 30° head-up positioning immediately after the procedure, a manoeuver usually performed to optimize hemostasis at the RIJ puncture site. Finally, the absence of a patent foramen ovale (or other right-to-left shunt) explains the venous—rather than arterial—cerebral distribution of air.

This case highlights potential preventive strategies when using the RIJ approach for LP implantation. Strict adherence to meticulous flushing and sheath preparation is essential. Adequate pre-procedural hydration and maintaining a slight Trendelenburg position during and immediately after the procedure may increase central venous pressure and reduce the risk of air aspiration. Systematic TTE at the end of the procedure to screen for residual intracardiac air could also be considered, potentially avoiding early patient verticalization and secondary air migration.^10^ Venous closure devices are sometimes used for large-bore venous access. However, their role in preventing air embolism—particularly in jugular access—remains unproven. In particular, implantation of a venosu closure device require additional manipulations at the access site, which may further expose the jugular vein to ambient air and therefore potentially favour air entrainment into the venous circulation.

## Patient’s perspective

While symptomatic air embolism is rare, its occurrence can lead to irreversible consequences. The reporting of this event contributes to greater awareness of a potentially under-recognized risk associated with the RIJ approach for LP implantation. Strict implementation of comprehensive preventive measures is crucial when performing LP implantation via the RIJ approach, to ultimately reduce the likelihood of similar events in future patients.

## Data Availability

The data underlying this article will be shared upon reasonable request to the corresponding author.

## Lead author biography

Dr Pierre Ollitrault is a senior cardiac electrophysiologist in the Department of Cardiology at Caen University Hospital Centre, Normandy, France. He is a fellow from the EHRA and the ESC.

## Notes

**Consent:** Written informed consent for scientific use of the data was obtained from the patient.
